# The β Form in PVDF Nanocomposites with Carbon Nanotubes: Structural Features and Properties

**DOI:** 10.3390/polym15061491

**Published:** 2023-03-16

**Authors:** María L. Cerrada, Javier Arranz-Andrés, Alicia Caballero-González, Enrique Blázquez-Blázquez, Ernesto Pérez

**Affiliations:** Instituto de Ciencia y Tecnología de Polímeros (ICTP-CSIC), Juan de la Cierva 3, 28006 Madrid, Spain; jarranz@csic.es (J.A.-A.); aligeminis9@gmail.com (A.C.-G.); enrique.blazquez@ictp.csic.es (E.B.-B.); ernestop@ictp.csic.es (E.P.)

**Keywords:** PVDF, CNT, nanocomposites, β and α polymorphs, conductivity, percolation

## Abstract

Different amounts of carbon nanotubes (CNT) have been incorporated in materials based on poly(vinylidene fluoride) (PVDF) by solvent blending followed by their further precipitation. Final processing was performed by compression molding. The morphological aspects and crystalline characteristics have been examined, additionally exploring in these nanocomposites the common routes described in the pristine PVDF to induce the β polymorph. This polar β phase has been found to be promoted by the simple inclusion of CNT. Therefore, coexistence of the α and β lattices occurs for the analyzed materials. The real-time variable-temperature X-ray diffraction measurements with synchrotron radiation at a wide angle have undoubtedly allowed us to observe the presence of the two polymorphs and determine the melting temperature of both crystalline modifications. Furthermore, the CNT plays a nucleating role in the PVDF crystallization, and also acts as reinforcement, increasing the stiffness of the nanocomposites. Moreover, the mobility within the amorphous and crystalline PVDF regions is found to change with the CNT content. Finally, the presence of CNT leads to a very remarkable increase in the conductivity parameter, in such a way that the transition from insulator to electrical conductor is reached in these nanocomposites at a percolation threshold ranging from 1 to 2 wt.%, leading to the excellent value of conductivity of 0.05 S/cm in the material with the highest content in CNT (8 wt.%).

## 1. Introduction

The development of a specific crystalline lattice plays a key role in semicrystalline polymers presenting different polymorphs, which can be formed depending mainly on the crystallization and processing conditions [1,2,3]. The intrinsic characteristics of a particular crystalline cell can deeply affect the whole spectrum of its properties. Poly(vinylidene fluoride) (PVDF) is a perfect example of that structural importance. This polymer shows steady electric polarization (ferroelectricity), which can be altered by the application of a mechanical field (piezoelectricity) and by a variation of temperature (pyroelectricity). This piezo- or pyroelectricity strongly depends on the type of polymorph developed during its preparation and on its content. Several crystallographic modifications have been found in PVDF with different polarity features, which involve three different chain conformations: TTTT for β phase, TGTG′ for α and δ lattices, and TTTGTTTG′ for γ and ε forms [4,5,6]. Despite the fact that each PVDF polymer chain shows an effective molecular dipole moment, only the β and γ phases have a dipole moment in the crystalline state. The former has aroused more technological interest because it provides the best pyro- and piezoelectrical performance. These electroactive polymorphs can be used in applications such as artificial muscles, actuators and sensors, acoustic transducers, medical imaging, wearable devices or smart textile, among others [7,8,9,10,11,12,13,14,15,16,17,18]. As a consequence, the increase of the content of the polar phases and suppression of the non-polar α phase, which is the easiest to obtain from melt and from solution, is of great importance for most of the applications of the PVDF-based materials.

The β lattice can be attained by means of different methods. The first route involves crystallization from the melt by applying an effective high cooling rate [19]. Nevertheless, the β modification usually coexists with the α form in these quenched samples [19,20], being the ratio between both the β and α lattices in the PVDF crystalline regions, dependent on the cooling rate. The pure β phase was achieved during PVDF crystallization from the melt at cooling rates above ca. 2000 K/s, as shown by chip calorimetry results in experiments performed using thin-films [21]. Application of even faster cooling rates (as high as 100,000 K/s) allowed for the obtainment of PVDF in a complete amorphous state [22]. A more recent study [23] pointed out that the α polymorph crystallized alone below 400 K/s while the β modification began to develop at that rate and at faster rates during non-isothermal crystallization experiments. A total of 50% of each phase was obtained at an approximate rate of 1100 K/s, as deduced from the variation of the percentage of each phase as a function of the cooling rate. Isothermal crystallization showed the appearance of three distinct regions [23]: up to a crystallization temperature (T_c_) of approximately 60 °C, the crystallization time increased exponentially with the temperature and corresponded to the formation of the β phase; afterward, there was an intermediate zone, approximately between 65 and 75 °C, where both crystalline modifications coexisted; and, a third region was observed at higher temperatures, where only α crystals were developed. Obviously, the cooling rate to reach the isothermal crystallization temperatures for observing the β phase must be high enough to avoid the formation of the α crystals on the way down.

Crystallization from the solution also allows development of the β form, which leads to coexistence, sometimes, with the α polymorph, depending on the experimental conditions applied [24,25,26]. Moreover, transformation from the α phase to the β modification by mechanical stretching is another route explored [27,28] together with the use of the electrospinning technique [29,30]. The β lattice development has also been described to be promoted by means of blending of PVDF with other miscible polymers, such as poly(methyl methacrilate) (PMMA) [31,32,33] or poly(1,4-butylene succinate) (PBS) [34] and by incorporation of nanofillers that might have interactions with PVDF, such as nanoclays, carbon nanofibers (CNF), carbon nanotubes (CNT), graphene oxide (GO), or cellulose nanocrystals (CNC) [29,35,36,37,38,39,40], among others. The protocol followed during the addition of the different components and the further processing are also very important for the formation of the β phase. For instance, nanocomposites of PVDF and CNT have been reported, sometimes, to lead to the pure α phase without any evidence of the β polymorph [41]. The applications in different fields such as energy storage and biomedical engineering have been described in the literature [42,43,44,45] for these materials.

The aim of this investigation is, first, to propose an easy and environmentally friendly methodology for the incorporation of CNT into PVDF that is able to boost the development of the β polymorph, and second, to proceed to its characterization in the resultant nanocomposites and to analyze their final electrical performance. CNTs tend to form dense aggregates or bundles in polymeric matrices due to their strong intrinsic Van der Waals forces, leading to their poor distribution. Thus, the development of an efficient protocol that allows the correct dispersion is essential. In addition, this procedure should be as environmentally harmless as possible. A solution blending method has been employed for the incorporation of different amounts of CNT into the PVDF matrix. Later, precipitation is selected [46] as the second preparation stage instead of evaporation, because the former allows for the recovery of solvents for their further utilization and, therefore, the circularity of resources. Afterwards, films have been attained by compression molding in order to establish the influence of CNT content on the polymorphic forms developed in PVDF, which will be characterized by Fourier transform infrared spectroscopy (FTIR) and by X-ray diffraction (XRD), using either conventional or synchrotron radiation. The determination of the electrical response of these nanocomposites is also an important point of the investigation.

## 2. Materials and Methods

A commercially available PVDF, with the trade name of Kynar 741, kindly supplied by Arkema (Colombes, France), has been used in the present research.

The multiwalled carbon nanotubes (CNT) (Baytubes C150P) were kindly supplied by Bayer Material Science AG (Leverkusen, Germany). Datasheet information indicates that they are characterized by a purity higher than 95 wt.%, the number of walls is between 2 and 15, the outer mean diameter ranges from 13 to 16 nm, the inner mean diameter is 4 nm, the length is between 1 and 10 μm, and the bulk density is around 150 kg/m^3^. They were used as received.

Both the PVDF and CNT were dried before the preparation of the nanocomposites by being placed in an oven at 110 °C for 24 h.

Afterwards, the nanocomposites with several contents in CNT (0.5, 1, 2, 4 and 8% by weight) were prepared using the following solution-precipitation approach: an appropriate amount of CNT was dispersed in *N,N*-dimethylformamide (DMF) (Sigma-Aldrich (San Luis, MO, USA) for each composition. Simultaneously, a PVDF/DMF solution was prepared. Both dispersion and solution were stirred for 90 min at room temperature. Then, the suspended CNTs were added slowly into the polymer solution and the resultant PVDF/CNT/DMF dispersions were additionally stirred within an ultrasound bath for 2 h. The final product was achieved by precipitation in ethanol. Afterward, it was filtered and then dried under vacuum for 24 h at 100 °C.

These nanocomposites were subsequently processed as films by compression molding in a hot-plate Collin press (Collin GmbH, Maitenbeth, Germany). Initially, the material was maintained at a temperature of 200 °C and at a pressure of 30 bar for 5 min. Later, a cooling process, at a relatively fast rate of around 80 °C/min and at a pressure of 30 bar, was applied to the different nanocomposites from their molten state to room temperature. These films are named PVDF, KCNT05, KCNT1, KCNT2, KCNT4 and KCNT8, for the neat matrix and the nanocomposites with different CNT contents, respectively.

The morphological details of different cryofractured sections with distinct CNT contents of these nanocomposites were obtained by transmission electron microscopy (TEM). Measurements were performed at room temperature in a 200 kV JEM-2100 JEOL microscope (JEOL Ltd., Tokyo, Japan). Thin sections of around 40 nm were cut by cryo-ultramicrotomy (Leica EM UC6, Leica Microsystems GmbH, Wetzlar, Germany) at −120 °C and deposited in a holder.

The fracture surface in different sections of the films was evaluated by scanning electron microscopy (SEM) using a Philips XL30 microscope (Philips, Leuven, Belgium) The samples were coated with a layer of 80:20 Au/Pd alloy and deposited in a holder before visualization.

The IR spectra were obtained on the films using a Perkin Elmer FTIR spectrometer equipped with an ATR device (Perkin Elmer, Waltham, MA, US), the scanning interval ranged between 650 and 4000 cm^−1^. Four scans were accumulated for each specimen at a resolution of 4 cm^−1^.

Wide angle X-Ray Diffraction (WAXD) patterns were recorded at room temperature in the reflection mode, by using a Bruker D8 Advance diffractometer provided with a PSD Vantec detector (Bruker, Madison, WI, USA). Cu Kα radiation (λ = 0.15418 nm) was used, operating at 40 kV and 40 mA. The parallel beam optics were adjusted by a parabolic Göbel mirror with a horizontal grazing incidence Soller slit of 0.12° and LiF monochromator. The equipment was calibrated with different standards. A step scanning mode was employed for the detector. The diffraction scans were collected with a 2θ step of 0.024° and 0.2 s per step.

Real-time synchrotron experiments have been performed on beamline BM11-NCD at ALBA (Cerdanyola del Vallés, Barcelona, Spain) at a fixed wavelength of 0.1283 nm (9.6617 keV). An ADSC 210 detector (with a pixel size of 102.4 μm), placed approximately at 200 mm from the position of the sample, was used. The temperature control unit was a Linkam hot stage, connected to a cooling system of liquid nitrogen. Typically, the diffractograms were acquired every 12 s (9 s of acquisition and 3 s of waiting time needed to refresh the detector). The calibration of spacings was obtained by means of a high-crystallinity iPP specimen ([040] diffraction at 1.910 nm^–1^) and of a silver behenate sample (5.838 nm spacing in its first-order reflection). The program FIT2D (ESRF, Dr. Hammersley) was used to convert the initial 2D X-ray pictures into 1D diffractograms. Additionally, the normalization of these diffractograms to the intensity of the direct beam was performed as well as the background subtraction of the sample control unit. The final diffractograms are represented against the inverse scattering vector, *s* = 1/*d* = 2 sin θ/λ.

Calorimetric analyses were carried out in a TA Instruments Q100 (TA Instruments, New Castle, DE, USA) calorimeter connected to a cooling system and calibrated with different standards. The sample weights ranged from 6 to 8 mg and the heating rate used was 20 °C min^−1^. For crystallinity determinations, a value of 104.5 J g^−1^ was used as the enthalpy of fusion of a perfect crystalline material [47,48,49].

Viscoelastic relaxations were measured with a TA Q800 Dynamic Mechanical Thermal Analyzer (TA Instruments, New Castle, DE, USA), working in a tensile mode. The loss tangent, tan δ, of the different composites, were determined as a function of temperature over a range from –150 to 150 °C at fixed frequencies of 1, 3, 10, and 30 Hz, and at a heating rate of 1.5 °C/min. For this analysis, strips of 2.2 mm wide and 15 mm length were cut from the molded films.

The electrical properties were measured with a Novocontrol BDS system (Novocontrol Technologies GmbH & Co. KG, Montabaur, Germany) comprising a frequency response analyzer (Solartron Schlumberger FRA 1260) and a broadband dielectric converter with an active sample head. Gold disk electrodes (20 mm in diameter) were used in the dielectric measurements carried out at 25 °C in the frequency window 10^−2^ to 10^7^ Hz. The temperature was controlled by a nitrogen jet (QUATRO from Novocontrol) with a temperature error of 0.1 K during every single sweep in frequency. The equipment was adjusted with a 100 Ω calibration standard.

## 3. Results and Discussion

The CNTs are incorporated into PVDF, the polymeric matrix, by solution blending using DMF as the solvent, as mentioned. DMF is the solvent most commonly used, although sometimes dimethylacetamide is also employed [40,50], and, less often, mixed solvents, such as tetrahydrofurane/DMF, DMF/acetone or water/DMF, are also tested [51,52,53]. This first stage is followed by precipitation with a non-solvent, a dryness process in a desiccator and a further compression molding to achieve different films based on PVDF and CNT, containing up to 8 wt.% in the latest. Before analyzing which type of PVDF crystalline structure has been developed under these conditions and if any electroactive PVDF polymorphs (β or γ) have been attained, dispersion of CNT within the bulk of the nanocomposites is examined.

**Figure 1:**
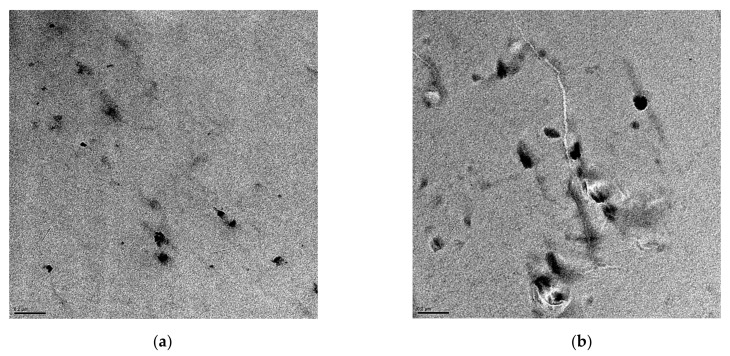
TEM micrographs of the (**a**) KCNT2 and (**b**) KCNT8 nanocomposites. The scale bar is referred to as 0.2 µm.

Figure 1 shows the TEM images for the KCNT2 (a) and the KCNT8 (b) nanocomposites. It can be observed that the CNTs are rather well distributed within the PVDF matrix, independently of its content. They can be found either as isolated units or as small aggregates, whose size is enlarged when increasing its amount in the nanocomposites.

The CNTs are able to establish strong intrinsic Van der Waals forces that promote the formation of aggregates or bundles in different polymeric matrices. Nevertheless, the conditions applied here allow a good distribution of these CNTs within the PVDF used, resulting in quite an effective procedure.

**Figure 2:**
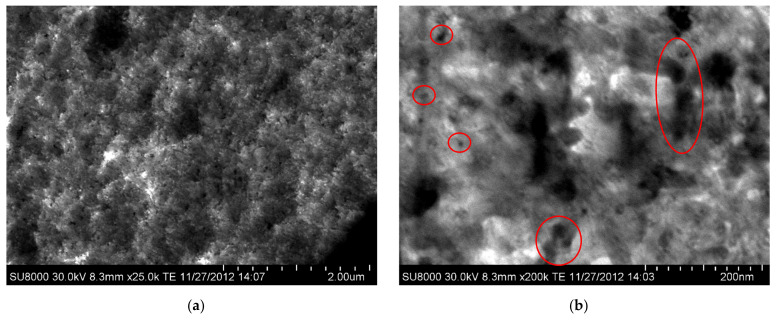
SEM micrographs of the KCNT2 nanocomposites at distinct augments: (**a**) scale bar of 2 µm; and, (**b**) scale bar of 200 nm.

This appropriate dispersion is also noticed in Figure 2, where the SEM images are shown for the KCNT2 nanocomposite at different augments. The CNTs can be clearly observed in their longitudinal and transverse sections. Again, it is noted that their aggregates are not of large dimensions.

**Figure 3:**
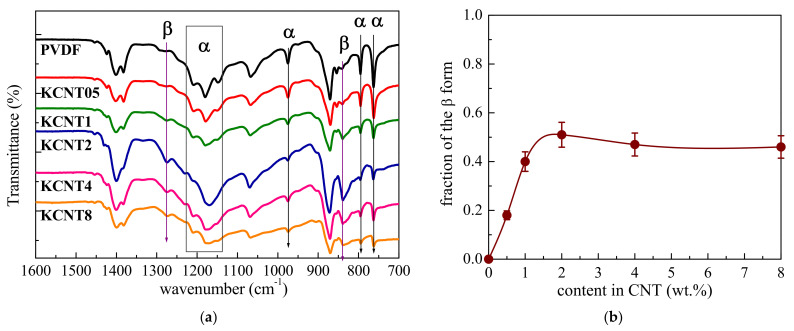
(**a**) FTIR spectra of the neat PVDF and the different nanocomposites under study. From top to bottom: PVDF, KCNT05, KCNT1, KCNT2, KCNT4, and KCNT8. (**b**) Fraction of the β polymorh in the several materials under study.

Now, once the dispersion of the CNTs has been learnt to be satisfactory in these nanocomposites, we turn to examine the crystalline lattice induced by the presence of these CNTs in the PVDF. Identification of the polymorphs of PVDF is commonly performed by FTIR and XRD measurements. The IR bands characteristic for the α phase appears at 764, 796, 976 and 1214 cm^−1^, while for the two main electroactive forms, i.e., for the β or γ phases, these bands are located at 840 and 1280 cm^−1^ in the former and at 812 and 1234 cm^−1^ in the latter, respectively [24,54,55]. Figure 3a shows the FTIR spectra for the PVDF and the different nanocomposites. The absence of the bands associated with the electroactive forms in the pristine PVDF reveals that this matrix, under the processing conditions applied, has crystallized only into the α lattice. The incorporation of a small amount of CNT in the KCNT05 material yields a small decrease in the intensity of the IR bands located at 764 and 796 cm^−1^ as well as that at 976 cm^−1^.

This reduction of intensity in these bands is more evident in the KCNT1 and becomes very noticeable in the other nanocomposites, i.e., in the KCNT2, KCNT4, and KCNT8. Simultaneously, the appearance of new bands, located at 840 and 1280 cm^−1^, starts to be observed as the CNT amount increases in these materials, indicating that the β polymorph has been developed along the processing. This cell is not the unique one since the characteristic bands of the α modification do not disappear completely but only decrease their intensity, a fact that points out the coexistence of both lattices within the PVDF crystalline regions in the nanocomposites.

The IR absorption bands at 763 and 840 cm^−1^ (specific of the α and β phases, respectively) are selected in order to quantify the fraction of the β form that has grown in each nanocomposite. Considering that IR absorption follows the Lambert−Beer law, the *A_α_* and *A_β_*, values of the absorbance at 763 and 840 cm^−1^ respectively, can be determined as:

(1) $$A_{\alpha }=\log\frac{I_{\alpha }^{0}}{I_{\alpha }}=K_{a}\cdot C\cdot X_{\alpha }\cdot L$$

(2) $$A_{\beta }=\log\frac{I_{\beta }^{0}}{I_{\beta }}=K_{\beta }\cdot C\cdot X_{\beta }\cdot L$$

where *L* is the sample thickness and *C* is an average total monomer concentration. The subscripts *α* and *β* are referred to as the two crystalline phases. *I*^0^ and *I* stand for the incident and transmitted intensity of the radiation, respectively; *K* is the absorption coefficient and *X* represents the amount of each phase. The *A_α_* and *A_β_* values are determined by *I*^0^ and *I* at 763 and 840 cm^−1^, respectively. *K_α_* and *K_β_* are the absorption coefficient of the respective bands [24] (*K_α_* = 6.1 × 10^4^ and *K_β_* = 7.7 × 10^4^ cm^2^/mol), and *X_α_* and *X_β_* are the content percentage of the respective phases. The relative *β* fraction, *F*(*β*) can be estimated [24] as:

(3) $$F(\beta )=\frac{X_{\beta }}{X_{\alpha }+X_{\beta }}=\frac{A_{\beta }}{(K_{\beta }/K_{\alpha })A_{\alpha }+A_{\beta }}=\frac{A_{\beta }}{(1.26)A_{\alpha }+A_{\beta }}$$

Figure 3b shows that the β lattice is not developed under the experimental conditions applied in the pure PVDF, as aforementioned. Consequently, only the α modification is then generated. The formation of this electroactive polymorph is favored by the presence of CNT, slightly in KCNT05, which is the nanocomposite with the smallest content, and in an increasing extent as its content is raised, reaching a plateau at around KCNT2. As already commented, this β phase always partially coexists with the α modification in the materials studied here, with a relative content of around 50% for the higher CNT contents.

**Figure 4:**
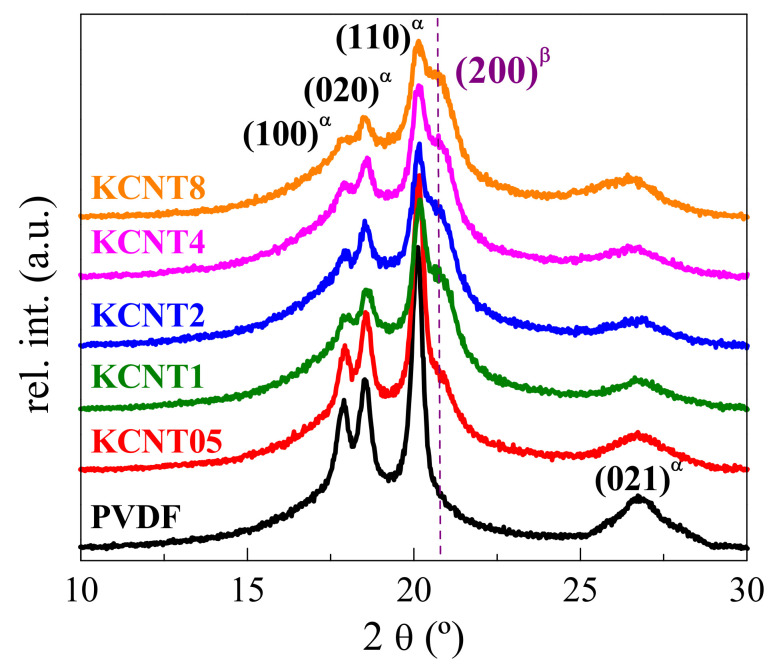
Room-temperature X-ray diffractograms of the neat PVDF and the different KCNT nanocomposites. The characteristic reflections of each crystalline phase are indicated.

Figure 4 shows the XRD profiles for the films of the neat PVDF and the different nanocomposites. As commented above, this technique is also very useful to detect the presence of the distinct cystalline lattices in this polymer and the materials based on it. The reflections of the α monoclinic form in the X-ray diffraction patterns are located at the values of angles 2θ of 17.7°, 18.5°, 19.9° and 26.5°, being ascribed to the planes (100), (020), (110), and (021), respectively. These are the diffractions that the pristine PVDF clearly shows since the monoclinic lattice is the only one that is developed in this matrix. On the other hand, the pure β modification in PVDF is characterized by a single reflection in this angular interval, appearing as a shoulder sometimes at positions ranging from 20.4 to 21.1°. In fact, it comes from the superposition of the (110) and (200) diffractions, and its location also coincides with the main diffraction of the γ crystal structure. Nevertheless, the absence of any specific IR band related to the γ form in the FTIR spectra, represented in Figure 3a, rules out the presence of this polymorph in the materials processed under these applied conditions.

The β formation is gradual in these PVDF nanocomposites, as CNT is increased. Thus, KCNT05 shows the common characteristics of the α modification although the beginning of a shoulder is observed on the high-angle side of the (110) reflection. The intensity of this additional reflection, assigned to the formation of the β lattice, grows as the CNT content is increased in the nanocomposites simultaneously with the broadening and decrease in the intensity of the specific reflections from the α form. Therefore, it turns out that the presence of CNT promotes the β development. These results are different to those described in literature for materials based on PVDF and CNT, where thermal conductivity was intended to be increased [41]. In those composites, the monoclinic α phase was the only one observed, most likely because of the different conditions applied during the processing compared with the ones imposed here. In fact, small variations in the cooling rate along the film obtainment can lead to important differences since the formation of the β polymorph in the PVDF is boosted at the highest rates [19,20,21,22,23].

**Figure 5:**
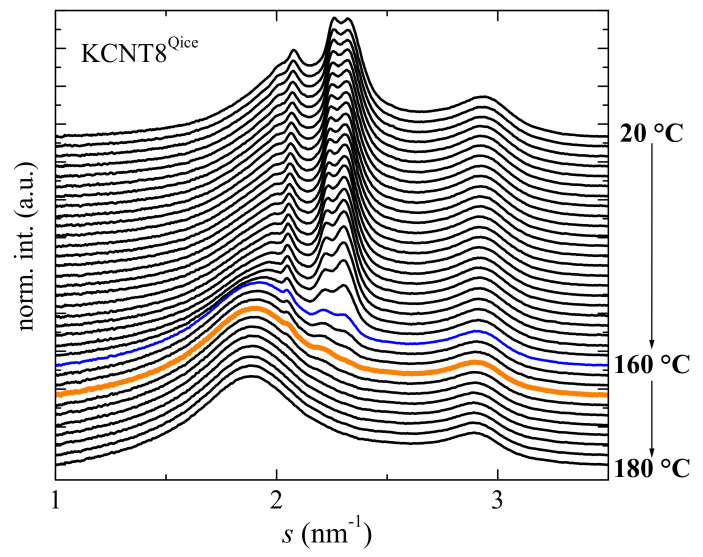
Real-time variable-temperature synchrotron profiles for the KCNT8^Qice^ specimen and their variation with temperature in the melting experiments at 20 °C/min. For clarity of the presentation, only one out of every three frames are plotted in the interval ranged from 20 to 160 °C, while all of the frames are represented from 160° to 180 °C.

Figure 5 shows the effect of two favorable parameters for the development of the β modification in the KCNT8 nanocomposite: the highest content in CNT and the application of a cooling rate faster than that used during the regular preparation of the film. This sample is referred to as KCNT8^Qice^. The results represented in Figure 5 are the ones attained from real-time variable-temperature X-ray diffraction measurements at wide angles (WAXS) with synchrotron radiation, corresponding to the melting of this sample. The KCNT8^Qice^ specimen was obtained from its rapid cooling from the melt by an immersion in an acetone/dry ice bath. Its profile at 20 °C, compared with that at room temperature found in the KCNT8 (see Figure 4), shows important differences: (a) the almost complete disappearance of the (100) reflection; (b) the split into two well-defined peaks for the (110)^α^ diffraction and the characteristic one from the β crystals (instead of its appearance as a shoulder, as in Figure 4). These features indicate that the relative fraction of the β polymorph regarding the α modification has increased in the KCNT8^Qice^ specimen because of the faster rate imposed during its cooling.

**Figure 6:**
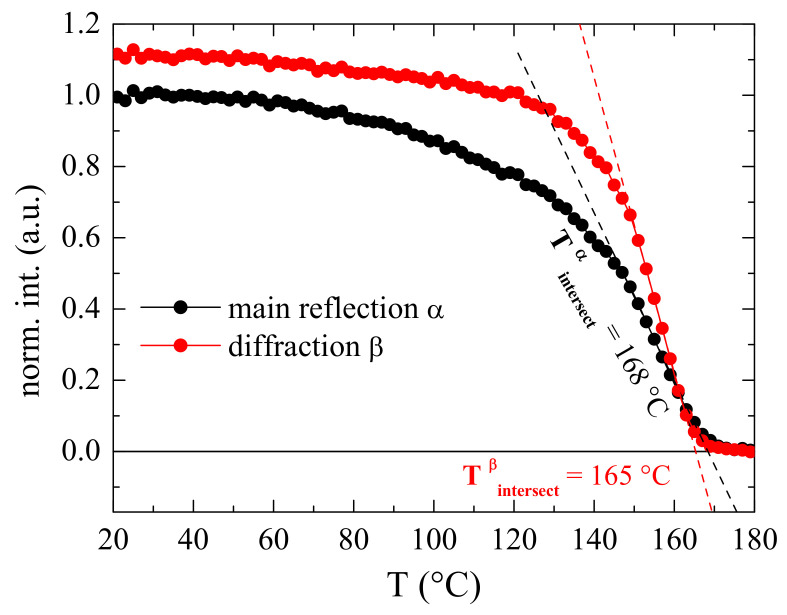
Dependence on temperature of the intensity for the main diffraction of the α phase and that characteristic for the electroactive β lattice. The intersection of the slope for their decrease at the higher temperature interval is an accurate indication of the T_m_ of both phases.

A detailed analysis of these X-ray patterns allows achieving information about the melting temperatures for these two crystalline phases, the α and the β, that coexist in this nanocomposite, as shown in Figure 6. In particular, the variation with temperature of their intensities has been evaluated. Initially, both characteristic diffractions show a rather similar intensity, as observed at room temperature in Figure 5. Then, there is a temperature interval, up to around 60 °C, at which both heights remain quite constant. At a higher temperature, the intensity of these two reflections begins to decrease but the α crystallites melt more significantly. Therefore, the intensities of the (020) and (110) diffractions diminish considerably while that for the reflection of β form is only slightly reduced (see Figure 5). This trend changes, however, at temperatures above around 140 °C where the β reflection is now the one whose height decreases sharper than those for diffractions of the α phase. Consequently, the β crystals melts at a temperature slightly lower than the α crystallites, as clearly noticed in the orange profile of Figure 5. The results represented in Figure 6 also allow quantifying the final melting temperatures (T_m_) of these two crystalline phases from the slope intersecting at a zero value of their heights. Accordingly, T_m_^β^ is 165 °C while T_m_^α^ is 168 °C.

**Figure 7:**
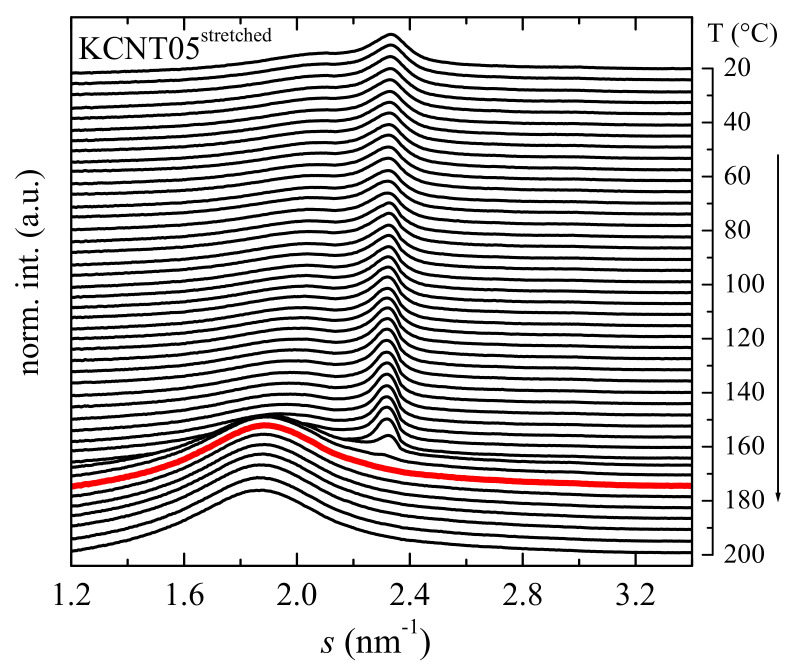
Real-time variable-temperature synchrotron profiles for the KCNT05^stretched^ sample and their variation with temperature in the melting experiments at 20 °C/min. Only one out of every two frames are plotted for clarity of the presentation.

Moreover, formation of the β polymorph has been also promoted by stretching in the KCNT05 nanocomposite, which is that with the lowest CNT content, crystallizing primarily in the α form, as deduced from the FTIR and XRD results represented in Figure 3 and Figure 4. Deformation was carried out at room temperature at a drawing ratio of 2. Figure 7 depicts that the KCNT05^stretched^ specimen only shows the β polymorph. Thus, the characteristic diffractions of the α modification are absent, this lattice being found mostly in the unstretched sample, as deduced from Figure 4. This β phase, developed partially from the α crystals together with that small amount of β crystallites initially existent in the KCNT05 sample, seems to be more perfect than that generated in the KCNT8^Qice^ nanocomposites since it melts at a slightly higher temperature (173 °C, red profile in Figure 7).

Another interesting aspect is related with the presence at 180 °C in the isotropic state, i.e., in the melt of the KCNT8^Qice^ of two amorphous halos. The one appearing at about 1.88 nm^−1^ is ascribed to the amorphous halo from the PVDF, while the one located at around 2.90 nm^−1^, which overlaps almost completely with the (021) diffraction of the α phase, is associated with the CNT. This second halo is not clearly observed in the molten state of KCNT05^stretched^ because of the small content in CNT that is incorporated in that material. Nevertheless, it can be slightly appreciated if the profiles are amplified.

Summarizing this section devoted to the crystalline structure, the incorporation of CNT into PVDF leads to the coexistence of the α and β polymorphs in these nanocomposites in different ratios depending upon the CNT content, the β phase being boosted as the CNT amount increases in the material. The β formation is also further favored by increasing the cooling rate during film processing, as shown in KCNT8^Qice^, and by stretching, as depicted in the KCNT05^stretched^ specimen. Identical approaches that promote the β modification in the neat PVDF seem to work in these nanocomposites. The extent is smaller in the former since the presence of CNT initially leads to the development of a partial fraction of the β polymorph.

**Figure 8:**
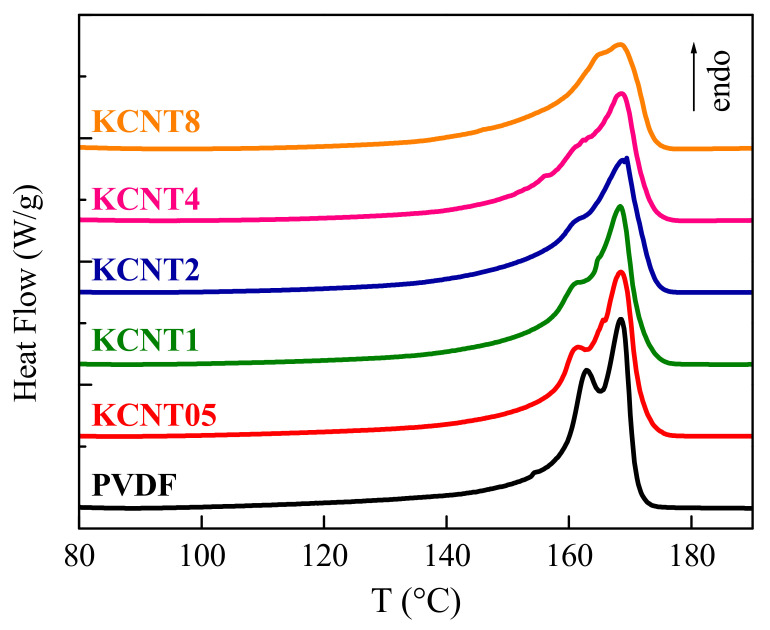
DSC curves obtained during the first heating run at 20 °C/min for the pristine PVDF and the different nanocomposites with CNT.

Figure 8 shows the DSC results for the phase transitions associated with the crystalline regions observed during the first heating run after processing for the pure PVDF and the different nanocomposites. A broad melting process is noted, which shows a bimodal shape very clearly in some of the materials and just as a shoulder overlapped with the main endotherm in the other cases. The cause of these multiple peaks is different in the neat PVDF than in the nanocomposites.

The behavior shown by the pristine PVDF, composed of two clear peaks, is often described in literature when an analogous thermal treatment has been employed during its processing [56]. The presence of these two peaks has been attributed to the melting-recrystallization phenomena, which are very significant at high temperatures, of the α crystallites existing in the neat PVDF. It is true that the existence of the two overlapped endotherms might also be associated with the melting of two distinct crystalline lattices or with the melting of two different crystallite populations. Nevertheless, those assumptions were rejected since: (a) on one hand, the unique crystalline form grown under the conditions applied was the monoclinic one (α crystallites), as demonstrated by both FTIR and real-time variable-temperature experiments with synchrotron radiation at wide angles. Additionally, (b) on the other hand, real-time variable-temperature measurements with synchrotron radiation at small angles did not point out the presence of two distinct crystallite populations. On the contrary, those results confirmed the improvement with temperature of the α crystalline structure through the melting-recrystallization processes [56]. The situation is more complex in the nanocomposites because of the coexistence of variable ratios of the α and β phases depending on the CNT. Therefore, the melting-recrystallization processes of both polymorphs can occur, together with the complete melting of the β form, which takes place at a lower temperature, as deduced in Figure 6, and the total melting of the α crystallites.

The T_m_ is not practically altered by the incorporation of CNT, independently of its amount, although the degree of crystallinity, which has been normalized to the actual PVDF content at each material, is reduced by increasing the CNT fraction present in the nanocomposite. This behavior could be ascribed to some hindrance for the three-dimensional ordering of the PVDF macrochains by the presence of CNT. Nevertheless, the final crystallites, although in a slightly smaller amount, are of similar size, showing an analogous T_m_, as deduced from Table 1. This trend is the opposite of that described in the materials that incorporated carbon nanofibers [57], where crystallinity and T_m_ increases in the nanocomposites in relation to the neat PVDF. Other systems using CNT, without and with a compatibilizer, show either an increase or a decrease in T_m_ and a confused dependence of the degree of crystallinity [41]. A rather constancy of T_m_ and an improvement in crystallinity was found in the nanocomposites with TiO_2_ [56]. Accordingly, the characteristics observed are dependent on the type of filler and also on the processing conditions.

**Figure 9:**
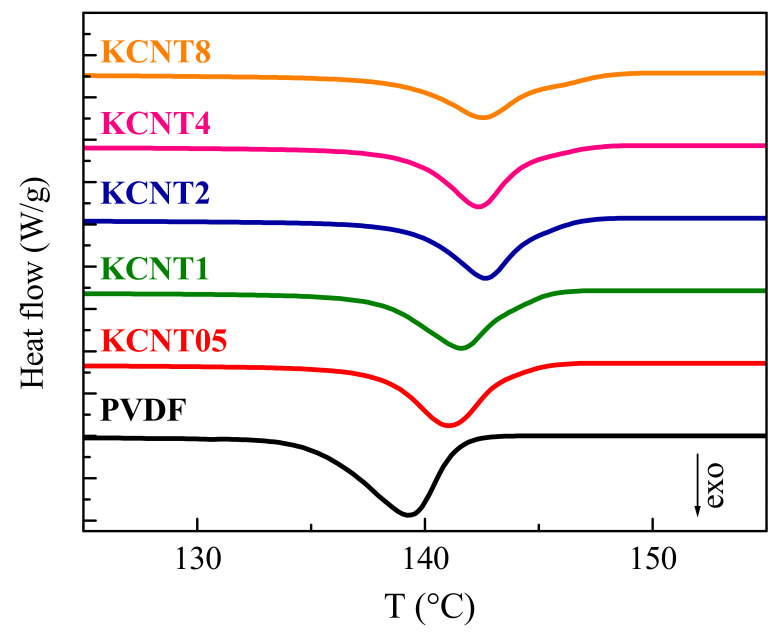
DSC curves obtained during the crystallization run at 20 °C/min for the pristine PVDF and the different nanocomposites with CNT.

Figure 9 shows the effect of the addition of CNT to the crystallization process of the PVDF. A clear nucleating effect is seen due to the presence of CNT. Accordingly, T_c_ is moved to higher temperatures than that for the neat PVDF. The location of T_c_ is shifted 2 °C as a very small amount is added in the KCNT05 material. At higher contents, the displacement increases slightly, reaching a plateau value for the higher contents, as reported in Table 1.

This nucleating influence of CNT has already been described in the literature, although there is no clear consensus. A small effect on the crystallization temperature of PVDF has been found up to 1 wt.% of loading [58], while further addition led to the increase of T_c_ from 141.6 °C to 143.7 °C. It was suggested that MWCNT acted as a nucleation agent for PVDF above a critical loading level. A very considerable nucleant role by the CNT was also reported in another investigation [41] and a displacement of T_c_ of almost 10 °C was stated.

**Figure 10:**
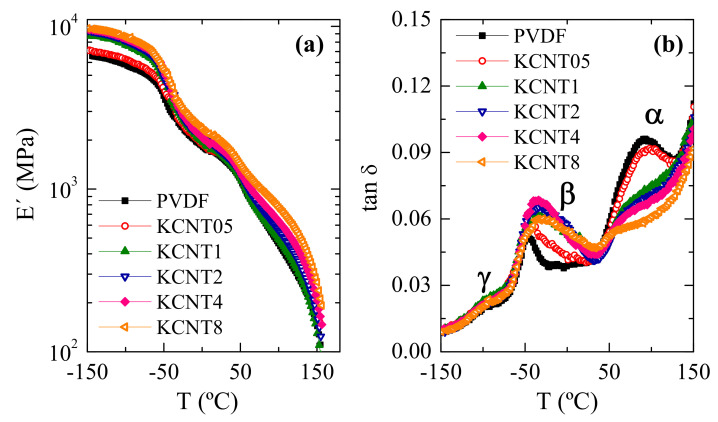
Dependence on temperature of: (**a**) the storage modulus (real component of complex modulus); and (**b**) loss tangent curves at 3 Hz of the neat PVDF and its KCNT nanocomposites with CNT.

Figure 10 shows the results attained from the dynamic mechanical thermal analysis related to the variation of the storage modulus (E′) and tan δ for the pristine PVDF and the distinct nanocomposites in a broad temperature interval, ranging from −150 to 150 °C. A dependence of the E′ upon the CNT content is noted in Figure 10a. This parameter provides us with an idea about the stiffness of these materials, and the values at 25 °C are the following: 1570, 1620, 1640 1690 1745 and 1940 MPa for PVDF, KCNT05, KCNT1, KCNT2, KCNT4, and KCNT8, respectively. They show a rather linear dependence of the CNT content, which confirms the good dispersion of the CNT within the several materials. The increase of rigidity with the CNT content observed in these nanocomposites is in agreement with the facts found previously in literature for PVDF with CNT [59] and with other fillers, such as Cu nanoparticles [49].

Figure 10b shows that three relaxation mechanisms are exhibited under tension at the temperature range analyzed for the PVDF and these nanocomposites, as clearly depicted in the tan δ representation. These relaxations are labeled as γ, β, and α in order of increasing temperatures [60,61,62]. The γ process, which is located at about −80 °C, has been attributed to molecular motions that take place in the amorphous regions. It is evidently superimposed with the β relaxation, which takes place at around −45 °C and is associated with the PVDF glass transition. The γ mechanism seems to be rather unaffected by the incorporation of CNT while the β one is more sensitive. Accordingly, intensity, width, and location of the maximum of the β relaxation is dependent on the CNT incorporation and on its content in these nanocomposites. The position of this transition has also been described to change in other materials with CNT [41,59]. Nevertheless, intensity and width were rather constant in PVDF-based materials where only α crystallites were developed [41,62]. In the KCNT composites under study, the coexistence of α and β crystals occurs as CNT are incorporated, in which the α/β ratio is reduced with the CNT content increasing to almost reach a plateau above 2 wt.%. This coexistence affects the amorphous regions and their mobility since the α and β crystallites are surrounded by them. The different characteristics of both crystalline polymorphs imposed distinct restrictions to the mobility of those chains in their amorphous state and, consequently, the width of the β relaxation (ascribed to the cooperative motions within the amorphous phase) is broadened. Concerning the intensity, the crystallinity is decreased as the CNT amount increases in the KCNT nanocomposites and, accordingly, the content of chains in the amorphous state is raised as well as its magnitude. Finally, the β relaxation is moved to higher temperatures because the presence of CNT makes the movements within the amorphous phase difficult and its location is shifted to higher temperatures: from −45.5 °C to −28 °C from the neat PVDF to the KCNT8 material. An important displacement of the β process was also reported in electrospun CNT reinforced PVDF fibers containing β crystallites [59].

The α mechanism, attributed to movements in the crystalline regions, is observed at around 90 °C in the pristine PVDF. A considerable decrease in the intensity of this relaxation is noted as the CNT concentration is raised in these KCNT nanocomposites together with a shift of its location to a lower temperature. Consequently, CNT plays a significant role not only in the mobility of the amorphous phase but also imposes motion restrictions within the crystalline regions.

**Figure 11:**
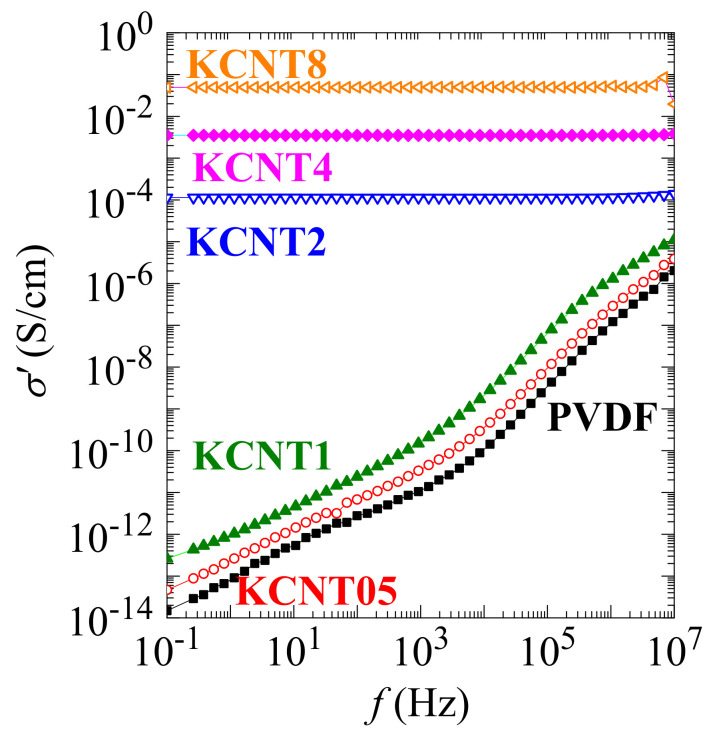
Electrical conductivity (σ′) measured at 25 °C as a function of frequency for the neat PVDF and the different KCNT nanocomposites with distinct contents in CNT.

Figure 11 shows the electrical conductivity (σ′) at room temperature as a function of frequency for pristine PVDF and the KCNT nanocomposites with different CNT contents. The KCNT05 and KCNT1 specimens exhibit a strong variation in their values of σ′ with frequency. Accordingly, σ′ rises as increasing frequency, which is a feature distinctive of insulating materials. This behavior is similar to that shown by the neat PVDF. Nanocomposites with higher amounts of CNT reveal greater values of σ′, increasing alongside the content. Furthermore, no frequency dependence is observed, i.e., the σ′ remains constant in the frequency interval employed. This remarkable enhancement in conductivity is well described by the percolation theory [63,64]. The KCNT nanocomposites are transformed from insulator into electrical conductor materials when the CNT concentration reaches a critical value, i.e., the percolation threshold. A conductive network is developed within the PVDF matrix, which induces this important improvement of conductivity in the vicinity of that percolation threshold, which is between 1 and 2 wt.% of CNT in these KCNT materials. The electrical conductivity raises about 10 orders of magnitude at a low frequency (10^−1^ Hz). Thus, the percolation threshold is a key parameter that highly influences the electrical properties of the composite materials. This high conductivity, along with the weak frequency dependence found in the KCNT nanocomposites, makes these materials excellent candidates, for example, for use in antistatic media and as shields against electromagnetic or radio frequency interference in electronic devices. 

If these values of conductivity are compared with others reported in the literature, it has to be mentioned that they are very remarkable. Thus, a value of around 7 10^−5^ S/cm has been described at a frequency of 10^0^ Hz for a nanocomposite containing a 5 wt.% of CNF [57] while values reached in chemically functionalized CNT composites were around 10^−3^ or lower depending on the type of modification [65]. Other authors earlier described values of conductivity of 10^−6^ S/cm at 100 Hz [66], presumed to be very good results, so those composites might be used to create some interesting devices, such as high charge-storage capacitors with various shapes. On the other hand, the highest conductivity was around 10^−4^ S/cm at 100 Hz in composites based on PVDF and hybrid CNT with BaTiO_3_ [67]. It can be concluded, therefore, that the conductivity values of the present nanocomposites are excellent, and one of the reasons may be the approach used for the preparation of the films, involving an initial protocol of solution/precipitation blending followed by compression molding.

## 4. Conclusions

Nanocomposites based on PVDF and different amounts of CNT (KCNTs) have been attained through solvent blending/precipitation and further processing by compression molding. With this procedure, the CNTs are found to be randomly dispersed within the polymeric PVDF matrix with the absence of noticeable domains of CNT with a great size across the nanocomposites, as revealed by the TEM and SEM observations.

Incorporation of the CNT into the PVDF promotes the crystallization of the β phase in the nanocomposites. Consequently, the crystalline structure observed in the neat matrix is completely of the α type, while coexistence of the α and β polymorphs is observed in the KCNT materials, with a β/α ratio that increases as the CNT content rises in the nanocomposite. This β polymorph is additionally induced in KCNT8 by cooling at a higher rate than that applied during the compression molding, and in KCNT05 by a uniaxial stretching at room temperature, up to a drawing ratio of 2. Real-time variable-temperature X-ray diffraction measurements with synchrotron radiation at wide angles (WAXS) undoubtedly allows observing the presence of the two polymorphs and determining the melting temperature of both crystalline modifications.

The CNT plays a nucleating role in the PVDF crystallization, displacing this exothermic process to higher temperatures on the cooling DSC experiments. Nevertheless, the degree of crystallinity is lowered as the CNT content increases in the nanocomposites. In spite of this crystallinity reduction, the addition of CNT leads to a reinforcement effect, and the stiffness in the nanocomposites is higher than that observed in the neat PVDF matrix. CNT incorporation also alters the mobility within the amorphous and crystalline PVDF regions, as deduced from the viscoelastic relaxations.

Finally, the presence of CNT leads to a very outstanding increase in the conductivity parameter, in such a way that the transition from insulator to electrical conductor is reached in these KCNT nanocomposites at a percolation threshold ranging from 1 to 2 wt.%. The value of conductivity is as high as 0.05 S/cm in the KCNT8 material, which is the one containing the highest amount in CNT. These optimal results may provide valuable applications for these nanocomposites.

## Figures and Tables

**Table 1:** Melting temperature (T_m_^F1^, first heating process) and crystallization temperature (T_c_); degree of crystallinity normalized to the actual PVDF amount (the first heating process as well as crystallization: f_c_^NORM^_F1_ and f_c_^NORM^_C_, respectively).

Samples	T_m_^F1^ (°C)	f_c_^NORM^_F1_	T_c_ (°C)	f_c_^NORM^_C_
PVDF	168.5	0.52	139.0	0.52
KCNT05	168.5	0.49	141.0	0.46
KCNT1	168.5	0.49	141.5	0.46
KCNT2	169.0	0.48	142.5	0.45
KCNT4	168.5	0.45	142.5	0.45
KCNT8	168.5	0.44	142.5	0.44

## Data Availability

Data are contained within the article.
